# Mid regional pro-adrenomedullin for the prediction of organ failure in infection. Results from a single centre study

**DOI:** 10.1371/journal.pone.0201491

**Published:** 2018-08-13

**Authors:** Bruno Viaggi, Daniele Poole, Omar Tujjar, Silvia Marchiani, Agostino Ognibene, Stefano Finazzi

**Affiliations:** 1 Dept of Anesthesia, NeuroIntensive Care Unit, Careggi University Hospital, Florence, Italy; 2 Anesthesia and intensive care operative Unit, S. Martino Hospital, Belluno, Italy; 3 Dept of Anesthesia, Salt University Healthcare Group, Sligo University Hospital, Sligo, Ireland; 4 General Laboratory, Careggi University Hospital, Florence, Italy; 5 GiViTI coordinating center, IRCCS, Istituto di Ricerche Farmacologiche “Mario Negri”, Ranica, Bergamo, Italy; International University of Health and Welfare, School of Medicine, JAPAN

## Abstract

Biomarkers are widely used to confirm the presence of infection. However, it would be of the greatest importance to predict in advance the occurrence or worsening of organ dysfunction in infected patients allowing timely antibiotic escalation. This study investigates the ability of procalcitonin (PCT) and MR-proADM to predict the transition to sepsis in infected patients. The study was conducted in a neurointensive care unit over a three-month period. We included both patients with and without infection to investigate the specificity of organ dysfunction prediction in infected patients. Daily measurement of PCT and MR-proADM, SOFA, Pitt, and CPIS were performed. To measure the correlation between each biomarker and each severity score, linear mixed-effects models were developed. For each biomarker-score combination we tested the correlation of the score with the biomarker measured one and two days before, the same day, and the day after. Sixty-four critically ill patients, 31 with infection, were enrolled. The statistically significant biomarker-score combinations were PCT-SOFA, MR-proADM-SOFA, MR-proADM-Pitt, and MR-proADM-CPIS. The MR-proADM models predicting Pitt and CPIS variations with 24-hour anticipation showed the best fit. The scores increased by 0.6 ± 0.3 and 0.4 ± 0.2 for each unitary biomarker increase, respectively. The MR-proADM-SOFA combinations were equivalent when the biomarker was measured the day before or the same day (score increases were 1.5 ± 0.4 and 1.9 ± 0.4, respectively). The PCT-SOFA model had the best fit when PCT was measured the same day of the score. There was no difference in the predictive ability of the biomarker in infected and non-infected patients. This was a pivotal study conducted in a single neurointensive centre on a limited number of patients, and as such it does not provide definitive conclusions. PR-proADM predicted occurrence and worsening of organ failure in critically ill patients with and without infection. The combination with infection diagnostic biomarkers such as PCT would allow predicting evolution to sepsis in infected patients.

## Introduction

The use of biomarkers has reshaped treatment and diagnostic strategies for sepsis. Among the others procalcitonin (PCT) has spread in clinical practice to support the diagnosis of infection, although cut-offs may vary according to the site of infection and the causative microorganism [1]. Moreover, the monitoring of PCT clearance guides antibiotic discontinuation, reducing the duration of treatment without increasing mortality [2, 3]. In recent years the mid-regional pro-adrenomedullin (MR-proADM), the 45–92 amino-acid sequence of adrenomedullin (ADM), has been proposed as a marker of infection alone or in combination with PCT [4]. MR-proADM has been identified as a stable ADM surrogate marker, whose assay is virtually inaccessible to any direct immunometric method, due to its rapid plasma clearance (about 22 min) and elevated protein binding. ADM is a peptide of 52 amino acids, it is detectable in plasma, widely distributed in different tissues, and secreted by endothelial cells and smooth vasal musculature. ADM is involved in systemic circulation control and performs an autocrine/paracrine vasoactive action, representing a key mediator of vasodilatation and playing a central role in the up/down regulation of most sepsis mediators and cytokines. As PCT, MR-proADM has been proposed to support the diagnosis of infection and as a predictor of mortality in infected patients [4–9]. Moreover, both MR-proADM and PCT have been shown to discriminate between infected patients with and without organ dysfunction [6, 10, 11]. These studies investigated the correlation of biomarkers measured the same day of organ dysfunction occurrence or worsening, thus with a diagnostic purpose. The objective of our study, instead, was to assess the ability of MR-proADM and PCT to predict clinical deterioration anticipating organ dysfunction variation. The nature of the study was pivotal, aimed at generating hypotheses in unexplored fields of application of MR-proADM.

## Methods

This prospective pivotal observational study was conducted in the Neuro-Intensive Care Unit of the Orthopaedic Trauma Center (CTO), at Careggi University Hospital, Florence. The local Ethical Committee (Comitato Etico Area Vasta Centro–Azienda Ospedaliera Universitaria Careggi) approved the study, and informed written consent to participate was obtained from all patients or their next of kin. The study was performed in accordance with the ethical standards of the 1964 Declaration of Helsinki and its later amendments.

### Study population

All patients with an infectious status encountered between the 12 of March and the 25 of June 2016 were included in an institutional database. Patients were excluded from the study if under 18 years of age, pregnant, or with end-stage diseases.

### Data collection

Data were collected prospectively using the Electronic Medical Record (EMR) NOME, developed by the Information Technology Service of the Careggi University Hospital. Relevant variables were extracted from EMR and all records were checked for completeness, accuracy, and consistency.

Demographics, pre-existing chronic diseases, type of admission (medical, surgical, or trauma), daily Sequential Organ Failure Assessment (SOFA)[12], Pitt[13] score, and Clinical Pulmonary Infection Score (CPIS)[14], as well as PCT and MR-proADM blood levels were collected daily in all patients. For patients in infectious status, results of microbiological tests (blood, urinary, sputum, bronchoalveolar lavage samples, and nasal and rectal swabs) were all recorded. Type, dose, duration, and route of administration of antimicrobial agents as well as treatments with vasoactive drugs, mechanical ventilation, and continuous renal replacement therapy (CRRT) were also noted, as were length of ICU stay and ICU and hospital outcome. Microbial identification was carried out with matrix assisted laser desorption ionization-time of flight mass spectrometry (MALDI-TOF MS), and pathogens’ sensitivity profiles to antimicrobials were determined with rapid molecular and phenotypic tests. MR-proADM was measured with the automated MR-proADM KRYPTOR immunoassay analyser.

### Definitions

Infectious status was defined by the attending physician’s clinical multiparametric evaluation, which included signs and symptoms of infection associated with indicative infection scores, positive plasma biomarkers, and/or microbiological results suggesting the presence of infection in a given sample.

For our analyses we could not adopt the sepsis 3 definitions [15], which are dichotomized according to a threshold of at least two SOFA points, because we needed the scores to be measured as continuous variables.

#### Statistical analysis

Descriptive statistics were computed for all study variables. Discrete variables were expressed as counts (percentage) and continuous variables as means ±SD or median (25th to 75th percentiles).

To determine the propensity of PCT and MR-proADM to diagnose or predict the modification of severity, we compared the values of each score (SOFA, Pitt, and CPIS) with the values of each biomarker. Since the biomarkers and the scores were measured daily, we could study the relation between the score value and the biomarker level being measured on the same day, two days before, one day before, and one day after (*shift parameter* 0, -2, -1, +1 days). Then, we developed linear mixed-effects (LME) models using the biomarker as the predictor (or independent variable) and the score as the outcome (or dependent variable). The LME is a linear regression model that measures the correlation between the biomarker and the score. However, compared to standard linear regression, LME has two advantages: first, it accounts for multiple measurements on the same subject; second, it accounts for differences among subjects in the relation between the biomarker and the score. Actually, in similar clinical conditions with similar evolutions, basal values and variations of biomarkers and scores may be quite different in different subjects. Averaging results, as in standard linear regression, in the case of such variability determines a substantial loss of information. LME, instead, allows measuring more reliable overall regression parameters, i.e. the average correlation between biomarkers and scores, accounting for individual variability.

We selected biomarker-score combinations (e.g. PCT-SOFA) for which slopes were statistically significant (i.e. significant *p* values for correlation between the biomarker and the score) but only for models with *shift parameter* 0, assuming that the best correlation was reached when the biomarker and the score were measured on the same day.

Then we compared all the LME models for each selected biomarker-score combination (thus, with *shift parameters* ranging from -2 to +1) with the Akaike Information Criterion (AIC), a statistical methodology that measures how well a model fits the data [16, 17]. The lower the AIC value, the better the fit, the more precisely the biomarker predicts the score (i.e. the two variables have the highest correlation). AIC values for each biomarker-score match were plotted against the *shift parameter* (from– 2 to + 1 days, by unitary steps) to illustrate which model fitted the best the data. Best fits occurring when the *shift parameter* is zero would indicate a diagnostic and confirmatory role of the biomarker. Best fits shown for negative *shift parameters* models would indicate that the biomarker has the highest correlation with the score measured on the following days, predicting clinical deterioration. In case the model with positive *shift parameters* best fitted the data, indicating a delayed correlation with the score, the biomarker would have no clinical usefulness. Consistently with rules of thumb reported in literature, delta AIC (e.g. AIC _shift parameter -1_ –AIC _shift parameter 0_) values ≤ 2 indicated absence of difference between the models, while higher differences indicated that the model, the one with the lower AIC value, fitted significantly better the data [17].

For the selected biomarker-score combinations we also reported in detail the statistics of models with *shift parameter* -1, because these models investigated the predictive ability of the biomarker to predict the score variation with a 24-hour anticipation. We did not test the other two models (*shift parameter*s -2 and +1).

For each model we calculated the ratio of the standard deviations of the random effects (i.e. the individual patient variability in the relation between the biomarker and the score) and the coefficients of the fixed effects (i.e. the intercept and slope which measure the overall relation between the biomarker and the score). This ratio is a measure of the variability of individual relations between the biomarker and the score. When the ratio is high it indicates that the spread of individual relations about the mean value is so large to advise against the use of overall coefficients (the general model intercept and slope) to predict individual organ dysfunction severity increases (measured by the score) on the basis of specific biomarker values.

Finally, for the selected biomarker-score combinations we tested the interaction between the presence of infection and the biomarker. The statistical difference between the log likelihoods of the models with and without the interaction was tested using the χ^2^ distribution. In the absence of statistically significant difference the most parsimonious models was preferred.

All statistical analyses were conducted using R version 3.3.3 [12].

## Results

The dataset used for our analyses is available as supplementary information (S1 Dataset). Of the 66 patients admitted to our ICU over the study period, 2 were excluded (early deaths), and 64 were included in the final cohort. The median age was 58 years, 36 (62.1%) were male, and 31 (48%) had an infection (Table 1). All infections occurred during the stay in the ICU, consistently with the case-mix of a neurointensive care unit, and were microbiologically documented.

**Table 1:** Characteristics of patients. Medians and interquartile range were calculated over all variables measurements. Q1 –Q3 = interquartile range PCT: procalcitonin; SOFA: daily Sequential Organ Failure Assessment; CPIS: Clinical Pulmonary Infection Score; GCS: Glasgow Coma Scale.

Age, median (Q1 − Q3)	58.0 (52.0 − 73.0)
Male n (%)	36 (62.1%)
Infected, n (%)	31 (48.4%)
Pneumonia n (%)	24 (37.5)
Primary bacteremia n (%)	6 (9.4)
Catheter related bacteremia n (%)	1 (1.6)
GCS, median (Q1 − Q3)	9 (5 − 11)
SOFA score, median (Q1 − Q3)	5 (4 − 8)
PITT score, median (Q1 − Q3)	4 (3 − 6)
CPIS score, median (Q1 − Q3)	3 (2–4)
PCT (ng/ml), median (Q1 − Q3)	0.3 (0.2 − 0.6)
pro-ADM (nmol/l), median (Q1 − Q3)	1.1 (0.8 − 1.7)

There were 649 measurements on 64 patients, with 48 missing values (7.4%).

The models analysing biomarkers and scores measured on the same day with statistically significant slopes were PCT-SOFA, MR-proADM-SOFA, MR-proADM-Pitt, and MR-proADM-CPIS (Table 2). For the same biomarker-score combinations slopes were also statistically significant for models with *shift parameter* -1, relating the score with biomarker measured one day earlier (Table 3). For each biomarker/score combination, models with *shift parameter* 0 included 649 measurements, while those with *shift parameter*—1, were 594 measurements since in this case the number of days of observation was less.

**Table 2:** Results of LME models with *shift parameter* 0: P-value of F-test on the slope parameters, AIC, ratios between the standard deviation of the random effect and the fixed effect of the intercept and the slope. All models have been developed using the same number of observations (649) grouped by patients. The AIC can be used to compare models with the same dependent variable. AIC: Akaike Information Criterion; LME: Linear Multiple-Effect; SOFA: daily Sequential Organ Failure Assessment; CPIS: Clinical Pulmonary Infection Score.

Dependent variable	Independent variable	F-test p-value	Slope (estimate ± SD)	random effect/ fixed effect	
				Intercept	Slope
SOFA Score	PCT	2.3 × 10^−4^	(1.5 ± 0.4) ml/ng	0.34	1.32
	pro-ADM	2.7 × 10^−5^	(1.9 ± 0.4) l/nmol	0.70	1.36
PITT Score	PCT	0.15	(1.9 ± 0.4) ml/ng	0.35	3.2
	pro-ADM	2.8 × 10^−3^	(0.7 ± 0.2) l/nmol	0.55	1.87
CPIS Score	PCT	0.15	(0.01 ± 0.008) ml/ng	0.39	0.005
	pro-ADM	0.02	(0.3 ± 0.1) l/nmol	0.50	1.73

**Table 3:** Results of LME models with *shift parameter* -1: P-value of F-test on the slope parameters, AIC, ratios between the standard deviation of the random effect and the fixed effect of the intercept and the slope. All models have been developed using the same number of observations (594) grouped by patients. The AIC can be used to compare models with the same dependent variable. LME: Linear Multiple-Effect; AIC: Akaike Information Criterion [18].

Dependent variable	Independent variable	F-test p-value	Slope (estimate ± SD)	random effect/ fixed effect	
				Intercept	Slope
SOFA Score	PCT	1.9 × 10^−4^	(1.4 ± 0.4) ml/ng	0.33	1.22
	pro-ADM	1.6 × 10^−4^	(1.5 ± 0.4) l/nmol	0.47	1.37
PITT Score	PCT	0.065	(0.2 ± 0.10) ml/ng	0.36	1.97
	pro-ADM	0.016	(0.6 ± 0.3) l/nmol	0.61	2.44
CPIS Score	PCT	0.12	(0.3 ± 0.2) ml/ng	0.45	3.03
	pro-ADM	0.038	(0.4 ± 0.2) l/nmol	0.62	2.25

**Fig 1:**
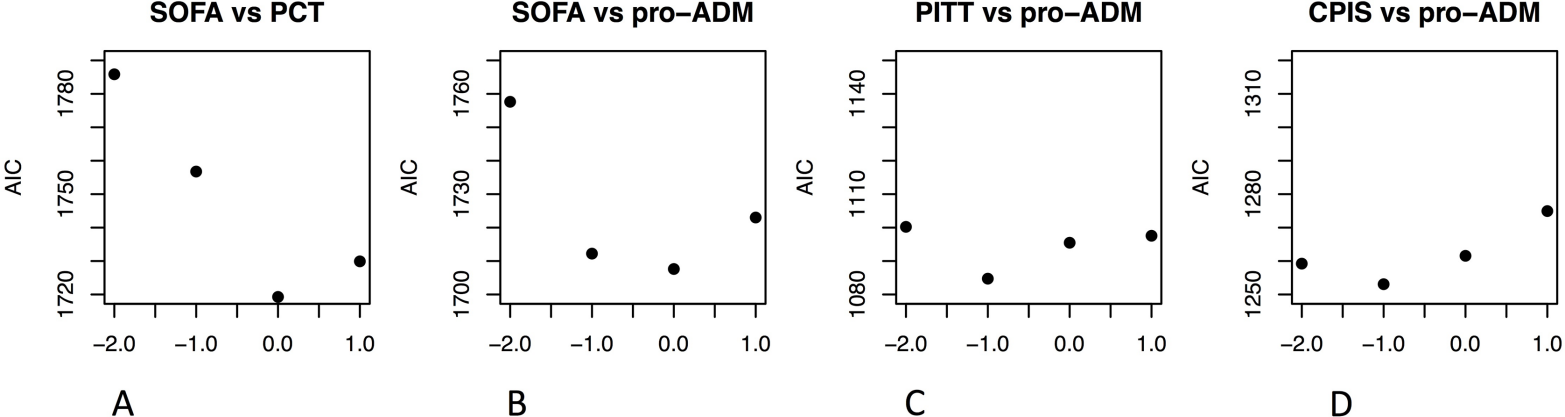
AIC of LME models with the biomarker measured two days before, one day before, the same day, the day after the score measurement (-2, -1, 0, and +1 on the x-axis). In panel A, we illustrate AIC values for LME models analyzing PCT and SOFA. In panels B, C, and D, the AIC values for the models correlating MR-proADM with SOFA, Pitt, and CPIS respectively. AIC: Akaike Information Criterion; LME: Linear Multiple-Effect; SOFA: daily Sequential Organ Failure Assessment; CPIS: Clinical Pulmonary Infection Score.

For each unitary increase of PCT concentration measured in ng/ml we found a 1.5 ± 0.4 SOFA increase when both were measured the same day. PCT anticipated a SOFA variation one day in advance with a 1.4 ± 0.4 score increase for each unitary PCT increase. The AIC was lower when the biomarker and the score were measured the same day (Fig 1) indicating a better fit of this model.

MR-proADM and SOFA, Pitt, and CPIS measurements performed the same day were correlated: for each unitary increase of MR-proADM concentration measured in mmol/l, scores increased by 1.9 ± 0.5, 0.7 ± 0.5, and 0.3 ± 0.1, respectively. MR-proADM also anticipated score variations with 1.5 ± 0.4, 0.6 ± 0.3, and 0.4 ± 0.2 score increases for each unitary increase of the biomarker, respectively.

AIC differences between models investigating the relation MR-proADM/SOFA measured on day -1 and 0 were small, while they were significantly higher for models where the biomarker anticipated the Pitt and CPIS compared to same days measurements (Fig 1).

Both PCT and MR-proADM were significant predictors of the SOFA score, but MR-proADM performed better than PCT, having a much lower AIC, with a delta AIC (AIC_PCT_−AIC_pro-ADM_) of 56.5.

In all the developed models, the ratio of random effects standard errors and fixed effects estimates, for both intercepts and slopes, ranged between 0.33 and 3.03.

There was no statistically significant difference between the models with and without the interaction with infection, with the exception of the CPIS-proADM combination (*p* < 0.001) where both the infection and its interaction with proADM were statistically significant (Tables A-D in the S1 Table).

## Discussion

Infection leads to a host inflammatory reaction aimed at confining the process locally eliminating the causal microorganisms. When the infection spreads beyond the local environment the systemic inflammatory response also becomes general, sometimes causing organ dysfunction and the transition from infection to sepsis with a striking increase in mortality[19, 20]. In the era of multidrug resistant microorganisms empiric treatment strategies of infections without organ dysfunction should spare life-saving molecules such as carbapenems. However, when infection evolves to sepsis (i.e. with infection related organ dysfunction) a more aggressive empiric approach is justified, because inappropriate therapy may be fatal for our patient[21]. In the past attempts have been made to predict the evolution of infection to sepsis and septic shock [20, 22]. In this context our findings are relevant, since they seem to indicate that MR-proADM predicts with 24-hour advance the occurrence or worsening of sepsis-related organ dysfunction, better than PCT, which was inferior in predicting SOFA variations and was not correlated with the Pitt and CPIS scores. The MR-proADM prediction models fitted the data better than the confirmatory models (i.e. biomarker and score measured the same day) for Pitt and CPIS, and equivalently for SOFA, suggesting that its predictive role could at least as valuable as its diagnostic ability. For the PCT-SOFA combination instead the best correlation was found for the confirmatory model, pointing in favour of its diagnostic role.

Interestingly, for the combinations of SOFA and Pitt with MR-proADM the models including the interaction between infection and the biomarker did not fit the data better than the more parsimonious models without the interaction, suggesting that organ dysfunction prediction was not specific but concerned all patients whether infected or not. For the CPIS-MR-proADM combination, instead, infection contributed significantly to the score increase and reduced the predictive weight of MR-proADM (Tables A-D in the S1 Table). This is not surprising since the CPIS is more focused on infection than on organ dysfunction compared to SOFA and Pitt and that most infections were ventilator associated pneumonias which is consistent with the findings of a recent report, that MR-proADM is a prognostic marker in community acquired pneumonia [23].

Thus, when dealing with organ dysfunction prediction in infected patients, a dual approach based on PCT for infection diagnosis and MR-proADM to monitor the evolution to sepsis could be hypothesized and would be consistent with physician’s clinical approaches. Actually, at bedside our diagnosis is never based on a single parameter but is the result of multiple data integration, which increases or decreases the likelihood of a specific diagnosis. The more diagnostic elements are available the highest the sensitivity and specificity of our approach, and the minimization of errors.

For methodological reasons we used the scores as continuous variables, thus a precise transition to sepsis according to current definitions was not measured and included in the analysis.

The lack of specificity of MR-proADM is not surprising. Actually, adrenomedulline has been shown to increase in several other diseases besides infections and its effects, such as vasodilation, bronchodilation, inotropism and diuresis increase, are not triggered specifically by bacteria [24].

An important result of our analysis is that we found a high ratio between random effects standard errors and the fixed effect estimate. Actually, although there is no precise cut-off for this ratio, when the dimension of the standard error is close to the dimension of the fixed effects estimate it can be certainly argued that variability is high. Transferred in a clinically meaningful concept this means that individual patients with similar clinical conditions and same risk of evolution to organ dysfunction may have very different serum concentrations of the biomarker, which hampers the definition of precise cut-offs needed in clinical practice. This finding, however, is not surprising since it is well known that studies investigating the diagnostic accuracy of PCT have provided very heterogeneous results, with PCT varying strikingly in relation to the infection site, the causative microorganism, and the severity of infection, and precise cut-offs for diagnosis could never be established[10, 25]. In our study the PCT ability to predict organ dysfunction was affected by the same large variability across individuals as MR-proADM. It is thus likely that MR-proADM may be influenced by other factors besides the worsening of organ function.

The main limitations are related to the neuro-intensive care setting where the study was conducted and the small sample size. The former influenced the type of infections we could deal with, limiting our case mix to pneumonia and bacteraemias either primary or catheter related, while abdominal and urinary infections were not included. This may be relevant because the response in terms of biomarker release may change in different organs. The latter, instead, may hamper the generalizability of our findings to other settings. Moreover, besides improving external validity, a multi-centre study with a larger sample would allow the development of more complex models, which could account for important influential variables and provide more precise cut-offs minimizing heterogeneity among individuals.

## Conclusions

Our study is a preliminary investigation conducted in a single neurointensive centre on a limited number of patients and it, hence, may have generalizability issues.

The ability of MR-proADM to anticipate the variation of several scores related to organ dysfunction is a finding that could have important implications for clinicians. However, since its predictive ability is not specific for infected patients a combination with a diagnostic biomarker as PCT, would be required to predict the evolution to sepsis or sepsis worsening.

## Supporting information

S1 Dataset csv file including the dataset used for the analyses. (CSV)

S1 Table Multilevel models including the interaction between biomarkers and infection (yes/vs. no). The ability of PCT and MR-proADM to predict SOFA variations on the same day, and of MR-proADM to predict Pitt and CPIS variations on the same, are reported in tables A, B, C, and D. PCT: procalcitonin; SOFA: daily Sequential Organ Failure Assessment; CPIS: Clinical Pulmonary Infection Score.
(DOCX)
