# Supplementary material for: Mid regional pro-adrenomedullin for the prediction of organ failure in infection. Results from a single centre study
Source: PLoS One. 2018 Aug 13;13(8):e0201491. doi: 10.1371/journal.pone.0201491 (PMC6089425; doi:10.1371/journal.pone.0201491)
Supplement: S1 Table — The ability of PCT and MR-proADM to predict SOFA variations on the same day, and of MR-proADM to predict Pitt and CPIS variations on the same, are reported in tables A, B, C, and D. PCT: procalcitonin; SOFA: daily Sequential Organ Failure Assessment; CPIS: Clinical Pulmonary Infection Score. (DOCX) [file pone.0201491.s002.docx]

| **Table A: PCT vs. SOFA** | | | | |
| --- | --- | --- | --- | --- |
|  | **Value** | **Std.Error** | **t-value** | **p-value** |
| Intercept | 5.47 | 0.30 | 18.30 | < 0.001 |
| PCT | 0.88 | 0.59 | 1.48 | 0.14 |
| Infection yes vs. no | -0.47 | 0.27 | -1.75 | 0.08 |
| Interaction | 1.14 | 0.57 | 2.00 | 0.05 |
| Statistical difference between the log likelihoods of the models with and without the interaction: *p* value 0.19 | | | | |

| **Table B: MR-pro-ADM vs. SOFA** | | | | |
| --- | --- | --- | --- | --- |
|  | **Value** | **Std.Error** | **t-value** | **p-value** |
| Intercept | 3.92 | 0.48 | 8.09 | < 0.001 |
| MR-proAMD | 1.69 | 0.46 | 3.67 | 0.00 |
| Infection yes vs. no | -0.37 | 0.30 | -1.26 | 0.21 |
| Interaction | 0.37 | 0.18 | 2.13 | 0.03 |
| Statistical difference between the log likelihoods of the models with and without the interaction: *p* value 0.41 | | | | |

| **Table C: MR-pro-ADM vs. PITT** | | | | |
| --- | --- | --- | --- | --- |
|  | **Value** | **Std.Error** | **t-value** | **p-value** |
| Intercept | 3.58 | 0.31 | 11.59 | < 0.001 |
| MR-proAMD | 0.67 | 0.23 | 2.91 | 0.00 |
| Infection yes vs. no | -0.10 | 0.15 | -0.66 | 0.51 |
| Interaction | 0.00 | 0.09 | -0.03 | 0.98 |
| Statistical difference between the log likelihoods of the models with and without the interaction: *p* value 1.0 | | | | |

| **Table D: MR-pro-ADM vs. CPIS** | | | | |
| --- | --- | --- | --- | --- |
|  | **Value** | **Std.Error** | **t-value** | **p-value** |
| Intercept | 1.99 | 0.20 | 9.95 | < 0.001 |
| MR-proAMD | 0.46 | 0.12 | 3.77 | < 0.001 |
| Infection yes vs. no | 1.09 | 0.16 | 6.90 | < 0.001 |
| Interaction | -0.30 | 0.09 | -3.36 | < 0.001 |
| Statistical difference between the log likelihoods of the models with and without the interaction: *p* value < 0.001 | | | | |
